# Pretreatment and enzymatic hydrolysis optimization of lignocellulosic biomass for ethanol, xylitol, and phenylacetylcarbinol co-production using *Candida magnoliae*

**DOI:** 10.3389/fbioe.2023.1332185

**Published:** 2024-01-18

**Authors:** Kritsadaporn Porninta, Julaluk Khemacheewakul, Charin Techapun, Yuthana Phimolsiripol, Kittisak Jantanasakulwong, Sumeth Sommanee, Chatchadaporn Mahakuntha, Juan Feng, Su Lwin Htike, Churairat Moukamnerd, Xinshu Zhuang, Wen Wang, Wei Qi, Fu-Li Li, Tianzhong Liu, Anbarasu Kumar, Rojarej Nunta, Noppol Leksawasdi

**Affiliations:** ^1^ Program in Biotechnology, Multidisciplinary and Interdisciplinary School, Chiang Mai University, Chiang Mai, Thailand; ^2^ Cluster of Agro Bio-Circular-Green Industry (Agro BCG), School of Agro-Industry, Faculty of Agro-Industry, Chiang Mai University, Chiang Mai, Thailand; ^3^ Faculty of Agro-Industry, Chiang Mai University, Chiang Mai, Thailand; ^4^ Guangzhou Institute of Energy Conversion, Chinese Academy of Sciences, CAS Key Laboratory of Renewable Energy, Guangdong Provincial Key Laboratory of New and Renewable Energy Research and Development, Guangzhou, China; ^5^ Key Laboratory of Biofuels, Qingdao Institute of Bioenergy and Bioprocess Technology, Chinese Academy of Sciences, Shandong Energy Institute, Qingdao New Energy Shandong Laboratory, Qingdao, China; ^6^ Department of Biotechnology, Periyar Maniammai Institute of Science & Technology (Deemed to be University), Thanjavur, India; ^7^ Division of Food Innovation and Business, Faculty of Agricultural Technology, Lampang Rajabhat University, Lampang, Thailand

**Keywords:** sustainability, ethanol, xylitol, phenylacetylcarbinol, optimization, response surface methodology

## Abstract

Cellulosic bioethanol production generally has a higher operating cost due to relatively expensive pretreatment strategies and low efficiency of enzymatic hydrolysis. The production of other high-value chemicals such as xylitol and phenylacetylcarbinol (PAC) is, thus, necessary to offset the cost and promote economic viability. The optimal conditions of diluted sulfuric acid pretreatment under boiling water at 95°C and subsequent enzymatic hydrolysis steps for sugarcane bagasse (SCB), rice straw (RS), and corn cob (CC) were optimized using the response surface methodology via a central composite design to simplify the process on the large-scale production. The optimal pretreatment conditions (diluted sulfuric acid concentration (% w/v), treatment time (min)) for SCB (3.36, 113), RS (3.77, 109), and CC (3.89, 112) and the optimal enzymatic hydrolysis conditions (pretreated solid concentration (% w/v), hydrolysis time (h)) for SCB (12.1, 93), RS (10.9, 61), and CC (12.0, 90) were achieved. CC xylose-rich and CC glucose-rich hydrolysates obtained from the respective optimal condition of pretreatment and enzymatic hydrolysis steps were used for xylitol and ethanol production. The statistically significant highest (*p* ≤ 0.05) xylitol and ethanol yields were 65% ± 1% and 86% ± 2% using *Candida magnoliae* TISTR 5664. *C. magnoliae* could statistically significantly degrade (*p* ≤ 0.05) the inhibitors previously formed during the pretreatment step, including up to 97% w/w hydroxymethylfurfural, 76% w/w furfural, and completely degraded acetic acid during the xylitol production. This study was the first report using the mixed whole cells harvested from xylitol and ethanol production as a biocatalyst in PAC biotransformation under a two-phase emulsion system (vegetable oil/1 M phosphate (Pi) buffer). PAC concentration could be improved by 2-fold compared to a single-phase emulsion system using only 1 M Pi buffer.

## Graphical Abstract

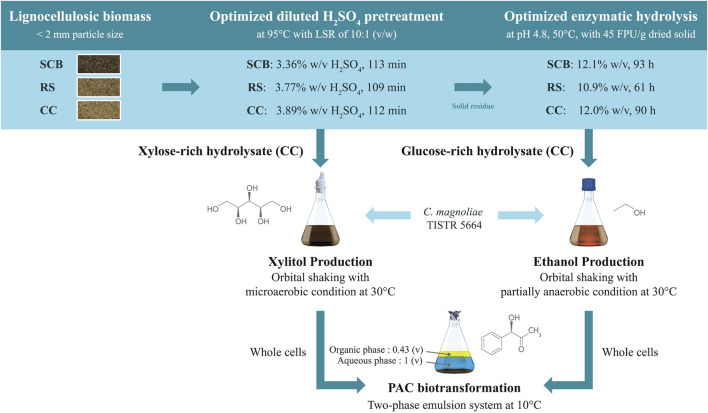

## 1 Introduction

An abundant quantity of agricultural waste biomass is being produced and used globally. The three crucial agricultural biomass in terms of availability are corn cob (CC), rice straw (RS), and sugarcane bagasse (SCB) with an overall worldwide production of 1.05 and 1.07 billion tons in 2019 and 2022, respectively. These could be compared with 50.9 million tons (4.85% of worldwide) and 30.6 million tons (2.87% of worldwide) in Thailand as of 2019 and 2022, respectively (OAE, 2023; Ritchie et al., 2023; USDA, 2023). These materials are commonly utilized for organic fertilizer, soil enrichment, and animal feed. Alternative usages of these wastes are also possible in biorefineries for renewable biochemical and bioenergy production (Galbe and Wallberg, 2019; Wang et al., 2022).

Lignocellulosic biomass is composed of carbohydrate polymers such as cellulose (38%–50%), hemicellulose (23%–32%), lignin (10%–25%), and a small amount of pectin, proteins, and extractives (chlorophyll, waxes, and non-structural sugars) (Bhatia et al., 2020; Bukhari et al., 2022). Cellulose is a homopolymer of glucose (6-carbon sugar or hexose sugar) monomers which are joined by *β*-1,4 linkages. Hemicellulose is a heteropolymer of 5-carbon sugar or pentose sugar (*β*-*d*-xylose, *α*-*l*-arabinose) and hexose sugar (*β*-*d*-mannose, *β*-*d*-glucose, *α*-*d*-galactose, and/or uronic acids) monomers by *β*-1,4 and *β*-1,3 linkages. Examples of relevant hemicellulose include xylan and glucomannan, with xylan being the most abundant (Kumar et al., 2020). Lignin is an amorphous irregular polymer with a complex three-dimensional network structure. Phenylpropanoid monomers comprised of phenylpropane units are generally found in this type of polymer. The three basic phenylpropane monomers of lignin structure are coniferyl alcohol (G type), sinapyl alcohol (S type), and *p*-coumaryl alcohol (H type) (Wang et al., 2023). Lignin severely limits the efficiency of enzymatic hydrolysis (Vermaas et al., 2015).

Pretreatment and enzymatic hydrolysis steps are two primary unit operations for several high-value biochemical production processes including bioethanol (Du et al., 2020), xylitol (Kim, 2019; Du et al., 2020), aldehydes, cellulose acetate, phenols, acids, and saccharides (Chen et al., 2017) from lignocellulosic materials. Diluted acid pretreatment appears to be a more favorable method for industrial applications as acid recovery step is not required (Refaat, 2012). The most common acid being used is sulfuric acid (H_2_SO_4_) which serves as catalyzer and dehydrating agent. Evidently, a higher monosaccharide conversion yield was also obtained when compared to another acids such as hydrochloric, phosphoric, and nitric acid (Chen et al., 2017; Khattab and Watanabe, 2019). It is generally utilized by bioethanol industrial plants in the United States (Nguyen et al., 2003), Brazil (Laranjeira et al., 2013), Russia (Lomovsky et al., 2009), and China (Rongjie et al., 2006). Under acidic conditions, the hemicellulose mass fraction could be hydrolyzed into monosaccharides rapidly with less than 10% w/w of remaining non-hydrolyzed solid. In fact, the cellulose and lignin fractions are relatively more stable and less susceptible to similar acidic conditions, possibly due to the absence of pentosan and xylan. The crystalline and amorphous without branched structures of cellulose may offer some resistance to diluted acid conditions (Chen, 2015; Galbe and Wallberg, 2019) while phenylpropanoid monomers in lignin can help facilitate protection against chemical and biological attacks (Lu et al., 2017). The accessibility of cellulose and hemicellulose to enzymatic hydrolysis can be enhanced through pretreatment processes with a relatively high yield of monosaccharides conversion (Kobkam et al., 2018). Under the 4%–8% w/v diluted sulfuric acid pretreatment step, a number of various inhibitors were formed in hydrolysate with relatively large quantities such as furfural, 5*-*hydroxymethylfurfural (HMF), and acetic acid at high temperature (Wang et al., 2019a). Furfural and HMF are the end products through the dehydration process of xylose or glucose, respectively, while acetic acid is formed primarily after the deacetylation step of xylan side chains (Davies et al., 2011). These inhibitors could slow down the microbial growth and, thus, decrease the overall fermentation performance (Wang et al., 2016). A detoxification strategy could be applied to mitigate the toxicity of these compounds using activated charcoal, ion-exchange resins, and over-liming with the side effect of sugars losses (Kaur et al., 2023). There existed several reports that implemented the intrinsic tolerance yeast strain such as *Candida tropicalis* with the capability of degrading inhibitors being formed in the pretreatment step (Cheng et al., 2014; Wang et al., 2016). The enzymatic hydrolysis step employs two principal enzymes, namely, cellulase and hemicellulase, which are commonly produced by various microorganisms. Cellulases hydrolyze *β*-1,4 linkages in cellulose chains, releasing oligosaccharides, cellobioses, and glucose molecules as the final product. This enzyme generally comprises three main units, namely, endoglucanase (EC 3.2.1.4), exoglucanase (EC 3.2.1.91), and *β*-glucosidase or cellobiase (EC 3.2.1.21). Xylanase is another primary hemicellulase capable of degrading the linear polysaccharide xylan into xylose by catalyzing the hydrolysis of the glycosidic linkage (*β*-1,4) of xylosides. The xylanase enzyme group comprises the xylanolytic enzyme system including endoxylanase (EC 3.2.1.8), *β*-xylosidase (EC 3.2.1.37), *α*-glucuronidase (EC 3.2.1.139), *α*-arabinofuranosidase (EC 3.2.1.55), and acetylxylan esterase (EC 3.1.1.72) (Liu and Kokare, 2017).

The hemicellulosic (xylose-rich) and cellulosic (glucose-rich) hydrolysates obtained from respective acid pretreatment and enzymatic hydrolysis steps could be utilized as carbon sources for xylitol and bioethanol production. Xylitol is a low-calorie sweetener, which is formed by the reduction of xylose through the activity of xylose reductase with either NADH + H^+^ or NADPH + H^+^ as co-factors. An NAD^+^-dependent xylitol dehydrogenase could then catalyze the subsequent conversion of xylitol into xylulose. After a further phosphorylation step, xylulose can enter the pentose phosphate pathway to produce ethanol (Mareczky et al., 2016; Baptista et al., 2018). Bioethanol is produced from acetaldehyde via the decarboxylation process of pyruvate from glucose with a subsequent reduction step together with NADH + H^+^ as a co-substrate under anaerobic conditions. This can be compared with the aerobic condition in which pyruvate is completely oxidized to CO_2_ through the Krebs cycle. Additional ATPs were obtained through oxidative phosphorylation in the mitochondria of several microorganisms (Pfeiffer and Morley, 2014). *Candida* spp. are well known for their ability to consume xylose for a relatively efficient production of xylitol and ethanol. *C. tropicalis* generally provides high production yields with resistance to non-detoxified hydrolysates as carbon sources (Cheng et al., 2014; Wang et al., 2016). *C. magnoliae*, an osmotolerant strain (Wannawilai and Sirisansaneeyakul, 2015), could be another interesting xylitol- and ethanol-producing yeast strain, as the statistically significantly similar (*p* > 0.05) xylitol and ethanol yields compared to the *C. tropicalis* strain were observed in our group.

The problem of relatively high cost of cellulosic bioethanol (USD 1.93–4.07/L) (Moonsamy et al., 2022) production stemmed from relatively expensive pretreatment and low-efficiency enzymatic hydrolysis steps is generally encountered. Although the ethanol production from the first-generation counterpart (molasses, sugarcane juice, and cassava hydrolysate) (USD 0.68–0.91/L) (Moonsamy et al., 2022) could be much cheaper, the disruption of food security in the supply chain is inevitable. The co-production of cellulosic bioethanol, xylitol (USD 8.66/kg) (IndiaMart, 2023b), and phenylacetylcarbinol (PAC) (USD 120/kg) (IndiaMart, 2023a)—a precursor of anti-asthmatic and nasal decongestant compounds, produced from pyruvate decarboxylase (PDC) (EC 4.1.1.1), a biocatalyst found in several ethanol-producing microorganisms (Leksawasdi et al., 2003; Leksawasdi et al., 2005)—was extensively studied to ensure economic viability and preserve PDC stability in a high-concentration phosphate (Pi) buffer (Khemacheewakul et al., 2018). Even though, there were studies which described high production of xylitol (>200 g/L) and/or ethanol (>100 g/L) such as the study by Meyrial et al. (1991) (221 g/L xylitol from *C. guilliermondii*), Ikeuchi et al. (1999) (256 g/L xylitol from *Candida* sp. 559-9), Cheng et al. (2010) (218.7 g/L xylitol from *C. tropicalis* in fed-batch fermentation), and Chang et al. (2018) (115 g/L ethanol from *S*. *cerevisiae*). These only reported the sole production of either xylitol or ethanol and were quite dissimilar from the current study, of which one yeast strain could be utilized for the subsequent production of three valuable compounds utilizing agricultural and agro-industrial wastes. Thus, the integrated high-value chemical production processes such as xylitol and PAC to the second-generation bioethanol production from the agricultural and agro-industrial wastes could have potential in offsetting the relatively high operating cost by fully utilizing the efficient yeast strain capable of producing these chemicals. In fact, yeast whole cells are usually considered a readily available by-product of the bioethanol production process at no cost that have not been utilized fully on the PAC production capability.

Therefore, this study aims to optimize and validate the diluted [sulfuric acid] pretreatment in boiling water and enzymatic hydrolysis steps for pulverized powder of SCB, RS, and CC based on the response surface methodology (RSM) in the xylose-rich and glucose-rich hydrolysate production for the selected lignocellulosic material. The xylitol and ethanol production by *C. magnoliae* TISTR 5664 were then compared in the absence of a detoxification step. The concentrations of important inhibitors were also monitored throughout the pretreatment and cultivation stages. Furthermore, the collected frozen–thawed whole cells from both xylitol and ethanol production steps were also subjected to PAC biotransformation in a two-phase emulsion system. The non-equivalent vegetable oil:1 M [Pi] buffer volume ratio of 0.43:1 was implemented. The novelty in co-produced processes of these chemicals in which the frozen–thawed whole cells from xylitol production was elucidated and applied for the first time in PAC biotransformation with significant improvement based on the two-phase emulsion system.

## 2 Materials and methods

### 2.1 Lignocellulosic materials

Lignocellulosic biomass, namely, SCB from Kaset Thai International Sugar Corporation and RS and CC from Chiang Mai Provincial Livestock Office, was used as substrates in this study. SCB was preliminarily prepared based on a method described by Nunta et al. (2023). RS was chopped into small pieces (about 2–5 cm). CC was ground in the first stage using a hammer mill machine (Champ AMCI Product, Thailand) with an 8 mm screen size. All materials were then subsequently attrited by using the hammer mill equipped with a 2 mm screen size (Ramaiah et al., 2020) before drying in a hot-air oven (LDO-100E, Lab tech, Korea) at 60°C for 24 h (Manorach et al., 2015). The pulverized powders of each lignocellulosic material were stored in sealed 50.8 × 76.2 cm^2^ polypropylene bags and kept in a dry place at room temperature until use. The proximate compositions of these powders (Table 1) were analyzed by Central Laboratory (Thailand) Co., Ltd., as elucidated in Analytical methods.

**TABLE 1:** Proximate analysis components of SCB, RS, and CC.

Component (% w/w, dry basis)	SCB	RS	CC
Ash	**12.7** ^**A**^ **± 0.6**	**14.0** ^**A**^ **± 0.2**	1.83^B^ ± 0.03
Carbohydrate^a^	77.0^B^ ± 1.8	73.5^B^ ± 0.3	**87.3** ^**A**^ **± 0.3**
Crude fiber	**31.6** ^**A**^ **± 0.8**	**31.6** ^**A**^ **± 0.6**	28.7^B^ ± 0.2
Fat	**2.33** ^**A**^ **± 0.15**	1.91^B^ ± 0.09	1.57^C^ ± 0.07
Protein	2.73^C^ ± 0.09	**5.10** ^**A**^ **± 0.12**	4.00^B^ ± 0.10
Moisture	**5.27** ^**A**^ **± 0.92**	**5.43** ^**A**^ **± 0.03**	**5.27** ^**A**^ **± 0.07**
Calories^a^ (kcal/100 g)	340^B^ ± 5	332^B^ ± 1	**380** ^**A**^ **± < 1**

^a^ Including dietary fiber.

Numbers with the same superscript capital alphabet indicate no significant difference (*p* > 0.05) for the comparison of the same row.

Bold values indicated the statistical significantly highest in the same row.

### 2.2 Commercial enzyme and chemicals

The commercial enzyme mixtures (Qingdao Vland Biotech Group Co., Ltd., Qingdao, China) were used to optimize the enzymatic hydrolysis of pretreated lignocellulosic biomass as described previously (Wattanapanom et al., 2019). The enzyme activity as the filter paper unit (FPU) and its specific activity were 103 ± 0.3 FPU/mL (Nunta et al., 2023) and 2.24 ± 0.06 FPU/mg protein, respectively. Additionally, the cellobiase activity unit (CBU) was also determined to be 2.568 ± 12 CBU/mL, with a specific activity of 55.7 ± 0.3 CBU/mg protein. All chemicals used in this study were analytical grade, excluding calcium hydroxide for pH adjustment of hydrolysates, which was commercial grade.

### 2.3 Microorganism

The microbial strain, *C. magnoliae* TISTR 5664 procured from the Thailand Institute of Scientific and Technological Research (TISTR), was propagated and stored at −20°C in the presence of 40% v/v glycerol stock (modified from the work of Nunta et al. (2019)).

### 2.4 Preparation of solid and liquid portions in pretreatment and enzymatic hydrolysis steps

The mixture of pulverized powder of lignocellulosic materials in the optimized concentration of diluted sulfuric acid was prepared. The diluted sulfuric acid (liquid)-to-powder (solid) ratio (LSR) was 10: 1 (v/w) (da Silva et al., 2015). The mixtures were then boiled at 95°C ± 1°C (Skiba et al., 2017) for an optimized time period suitable to the production scale. The liquid and solid portions of these hydrolysates were separated by the filtration technique using a two-layer muslin cloth (adapted from the work of Nunta et al. (2019)). The liquid portion could be used for xylitol production, while the solid was washed with running tap water until the pH level reached 4–5 (Lee et al., 2015) and then dried at 60°C to attain a constant weight (Manorach et al., 2015). The obtained solid was subsequently used in the further enzymatic hydrolysis step with an enzyme loading of 45 FPU/g dried solid (Dávila et al., 2016) in 50 mM Na-citrate buffer at pH 4.8 under 50°C under good mixing conditions (Manorach et al., 2015; Koti et al., 2016; Li J. et al., 2019). After the enzymatic hydrolysis step, the enzymatic denaturation was achieved by subjecting the mixture to a boiling condition of 95°C ± 1°C. After boiling, the mixture was cooled down to 10°C and the residual solid was removed with the appropriate separation technique (modified from the work of Qi et al. (2009)).

### 2.5 Cultivation media and microbial propagation

A yeast-malt medium supplemented with 5 g/L xylose (YMX) (Stoklosa et al., 2019) was used as a pre-seed and seed cultivation medium for microbial propagation. The glycerol stock of *C. magnoliae* TISTR 5664 was cultivated in the YMX medium at 30°C and 200 rpm for 24 h (Nunta et al., 2018) before using it as seed inoculum. The initial cell concentration was 1.58–1.67 × 10^8^ CFU/mL with cell viability above 99%. Xylose-rich and glucose-rich hydrolysates for xylitol and ethanol production were prepared based on optimal conditions for the pretreatment and enzymatic hydrolysis of the best lignocellulosic material. Xylose-rich hydrolysate was the liquid portion obtained after the pretreatment step using 1.5 L diluted sulfuric acid in 2 L laboratory glass bottles under boiling conditions in an autoclave (LAC-5100SD, Lab tech, Korea). Glucose-rich hydrolysate was the remnant liquid portion obtained after the enzymatic hydrolysis step. The preparation was conducted with a working volume of 25 L in a 50 L temperature-controlled mixing tank (MT001, FENIX International, Thailand) with a three-bladed propeller. The boiling time was 15 min, while centrifugation at 3,580 × g for 15 min was used as a separation technique. A further concentration step by vacuum evaporation using a rotary evaporator (R-1010, Greatwall, China) at 70°C (Unrean and Ketsub, 2018) was then applied for both hydrolysates to achieve the optimal xylose (50 g/L) and glucose (100 g/L) concentration for xylitol and ethanol production based on the previous literature (King and Hossain, 1982; Sirisansaneeyakul et al., 1995; Nurhayati et al., 2014; Chavan et al., 2015; Tangri and Singh, 2017; Saha and Kennedy, 2020). An antifoaming agent mixture of olive oil (0.1% v/v) and polysorbate (Tween 20) (0.01% v/v) was added to both hydrolysates before the evaporation step based on the final concentrated hydrolysate volume (adapted from the work of Ishwaryaa and Nisha (2021)). All hydrolysates were supplemented with ammonium sulfate at 8.52 g/L as a defined nitrogen source (Nunta et al., 2018) with pH adjustment to 6.0 using calcium hydroxide powder (Unrean and Ketsub, 2018). After pH adjustment, the centrifugation process was then followed at a similar condition once more to remove the calcium sulfate precipitate being formed during the pH adjustment step. The supernatants from both hydrolysates were subsequently kept at −20°C in 1.5 L polyethylene terephthalate bottles until use. All cultivation media were sterilized at 110°C for 20 min in the autoclave before cultivation (Kumar et al., 2018).

### 2.6 Experimental by a central composite design

A central composite design (CCD) with two variables, three levels, and five replicates at the center point was used for fitting a second-order response surface in the optimization of pretreatment and enzymatic hydrolysis. The variable ranges from the center point of the design space to a factorial point are ±1 unit for each variable. The axial points are at a distance of ± α from the center point (α = ± 1). Eq. 1 represents the quadratic model for predicting the responses.

$$Y=\beta_{0}+\sum \beta_{i}X_{i}+\sum \beta_{ii}X_{i}^{2}+\sum \beta_{ij}X_{i}X_{j},$$ (1)

where Y is the predicted response; β_0_ is a constant; β_i_ is the linear coefficient; β_ii_ is the squared coefficient; β_ij_ is the interaction coefficient; X_i_ is variable i; and X_j_ is variable j.

To correlate the response variable to the independent factors, the former was projected to experimental data using a predictive polynomial quadratic equation (Eq. 1). Design-Expert 6.0.2 (Stat-Ease, United States) was the statistical software employed for the regression procedure, graphical analyses, and computation of Fisher (F) test, analysis of variance (ANOVA), correlation coefficient (*R* ^2^), adjusted (Adj) *R* ^2^, and coefficient of variation (CV) values (Chaiyaso et al., 2011).

### 2.7 Optimization of pretreatment using the RSM via the CCD

The CCD with two variables of diluted sulfuric acid concentration and reaction time was used for this pretreatment optimization. The variable ranges were adapted from the work of Ren et al. (2015) and were assigned as (0.5, 2.75, and 5.0% w/v) and (30, 135, and 240 min), respectively. Table 2 shows both variables and the corresponding three levels with five replications of these variables being investigated. The experiments were carried out using the condition described in Preparation of solid and liquid portions in pretreatment and enzymatic hydrolysis steps with 100 mL diluted sulfuric acid in 250 mL non-baffled Erlenmeyer flasks. The dried pretreated solids of SCB, RS, and CC were measured for mass basis compositions of cellulose and lignin in solid residue (% w/w) responses after pretreatment.

**TABLE 2:** Experimental design and CCD responses for the optimization of the pretreatment step.

Run number	Variables (*X*)		Responses (*Y*)					
			SCB		RS		CC	
	*X*_1_	*X*_2_	*Y*_1,SCB_	*Y*_2,SCB_	*Y*_1,RS_	*Y*_2,RS_	*Y*_1,CC_	*Y*_2,CC_
1	0.50	30	60.3	***16*.*5***	***54*.*2***	***5*.*22***	***41*.*5***	***8*.*53***
2	5.00	30	63.8	18.0	67.4	7.98	57.6	11.3
3	0.50	240	64.1	18.6	57.3	5.96	53.9	10.4
4	5.00	240	**68.6**	**21.0**	**71.1**	**10.99**	**67.7**	**17.5**
5	0.50	135	***59*.*9***	16.9	56.0	5.97	47.8	***8*.*53***
6	5.00	135	67.4	19.3	71.2	9.11	65.9	15.0
7	2.75	30	64.1	17.8	62.2	6.69	56.5	11.1
8	2.75	240	66.8	19.8	69.0	9.53	65.3	14.7
9^a^	2.75	135	66.2	20.9	68.0	8.39	63.4	14.1
10^a^	2.75	135	66.1	19.1	68.7	8.69	64.2	13.6
11^a^	2.75	135	66.6	20.0	70.2	8.88	62.5	14.6
12^a^	2.75	135	66.7	19.3	68.8	8.37	63.1	14.6
13^a^	2.75	135	66.6	20.0	68.5	8.75	63.4	15.4
*p*-value			0.0002	0.0004	<0.0001	<0.0001	<0.0001	<0.0001
*R* ^2^			0.95	0.94	0.98	0.97	0.99	0.97
Adj-*R* ^2^			0.91	0.90	0.96	0.95	0.98	0.95
CV (%)			1.34	2.32	1.88	4.38	1.68	6.27

*X*_1_ = diluted [sulfuric acid] (% w/v), *X*_2_ = treatment time (min), *Y*_1_ = cellulose content in the solid residue (% w/w), *Y*_2_ = lignin content in the solid residue (% w/w). Bolded numbers indicate the highest values. Bolded, and italicized numbers indicate the lowest values.

^a^ Quintuplicate were applied at the center point.

### 2.8 Optimization of enzymatic hydrolysis using the RSM via CCD

Similar CCD optimization was also used in this process with three levels of [pretreated solid] (5, 12.5, and 20% w/v) and hydrolysis time (48, 144, and 240 h). The variable ranges were adapted from the work of Assabjeu et al. (2020), Phummala et al. (2015), and Qi et al. (2009) as tabulated in Table 3. The experiments were performed using the condition described in Preparation of solid and liquid portions in pretreatment and enzymatic hydrolysis steps with 25 mL working volume in 125 mL non-baffled Erlenmeyer flasks with a shaking speed of 200 rpm in a shaking incubator (LSI-3016R, Lab tech, Korea) and 3 min boiling time. Residual solids were obtained by the filtration technique using a two-layer muslin cloth (modified from the work of Nunta et al. (2019) and Qi et al. (2009)). The liquid portion was analyzed for sugar concentration to measure the response of glucose yield (% w/w) as estimated by Eq. 2 (adapted from the work of Qi et al. (2009) and Wang et al. (2019b)).

$$Glucose\;yield\;\left(\%\;w/w\right)=\frac{glucose\;\left(g\right)\times 0.9}{pretreated\;solid\;mass\;\left(g\right)}\times 100,$$ (2)

where 0.9 is the theoretical dehydration coefficient from glucose to cellulose as stated by Wang et al. (2019b).

**TABLE 3:** Model validation of the optimal pretreatment conditions with the corresponding predicted responses, actual responses, and relative errors of cellulose and lignin contents for SCB, RS, and CC.

Lignocellulosic material	Diluted [H_2_SO_4_] (% w/v)	Treatment time (min)	Type of responses	Predicted values (% w/w)	Actual values (% w/w)	Relative errors (%)
SCB	3.36	113	Cellulose	66.7	68.0 ± 0.3	1.88
			Lignin	19.7	19.5 ± 0.1	0.51
RS	3.77	109	Cellulose	70.1	68.2 ± 1.1	2.78
			Lignin	8.84	8.54 ± 0.09	3.48
CC	3.89	112	Cellulose	64.5	61.6 ± 0.8	4.73
			Lignin	14.5	16.0 ± 0.4	8.94

### 2.9 Validation of the RSM

The quadratic model (Eq. 1) for predicting three responses on mass bases (% w/w), namely, mass basis compositions of 1) cellulose and 2) lignin in solid residue after pretreatment, as well as 3) glucose yield, was confirmed for its validity by the assessment of relative error percentage (% RE) lower than 10% (Maupin et al., 2017). The optimal conditions suggested by the models were evaluated experimentally in triplicate.

### 2.10 Production of xylitol and ethanol

Shake-flask cultivations were performed with 100 mL working volumes in 250 mL non-baffled Erlenmeyer flasks with 10% v/v inoculation from the seed culture of *C. magnoliae* TISTR 5664. For xylitol production, xylose-rich hydrolysate prepared in gauze plug Erlenmeyer flasks was incubated at 30°C and 200 rpm in a shaking incubator without a pH controller under microaerobic conditions (adapted from the work of Antunes et al. (2021); Cheng et al. (2014); Zhao et al. (2013)). For ethanol production, glucose-rich hydrolysate in screw cap Erlenmeyer flasks was incubated under partially anaerobic conditions at 30°C with a rotation speed of 100 rpm (adapted from Cheng et al. (2014); Zhao et al. (2013)) without a pH controller. A total of 4 mL samples were collected from triplicate flasks at regular intervals from the beginning at every 24 h until 240 h. The related kinetic parameters were computed as indicated in Analytical methods.

### 2.11 PAC biotransformation in the two-phase emulsion system

The two-phase emulsion system using organic and aqueous phases with the optimal volume ratio of 0.43:1 (Gunawan et al., 2008) was carried out using the total combined volume of 25 mL in a 125 mL Erlenmeyer flask at 10°C (Tangtua et al., 2013). The frozen–thawed whole cells of *C. magnoliae* with an initial volumetric PDC activity of 1.53 ± 0.04 U/mL were used in the biotransformation. This system comprised vegetable oil as an organic phase with 200 mM benzaldehyde (Bz) and 1 M Pi buffer (pH 6.5/10 M KOH) as an aqueous phase with 240 mM sodium pyruvate (Pyr). These prescribed [Bz] and [Pyr] were as appeared in the total combined volume of both organic and aqueous phases. The concentration of co-factors, namely, thiamine pyrophosphate and magnesium sulfate heptahydrate, was 1 mM (Nunta et al., 2018).

### 2.12 Analytical methods

The proximate analyses of SCB, RS, and CC pulverized powder were conducted by Central Laboratory (Thailand) Co., Ltd., based on the method of analysis for nutrition labeling for carbohydrates and calories (Sullivan and Carpenter, 1993). The contents of fat (954.02), crude fiber (978.10), protein (981.10), ash (942.05), and moisture (930.15) were determined using AOAC methods (AOAC, 2019). The cellulase and cellobiase activities were evaluated as described by Ghose (1987) with similar definitions of respective enzyme activities. The protein concentration of commercial enzyme mixtures, cultivation media, and the aqueous buffer of the biotransformation system were analyzed by Bradford assay (Bradford, 1976). The compositions of cellulose, hemicellulose, and lignin in lignocellulosic biomass and the solid portion after the pretreatment step were determined by the sequential method of Van Soest et al. (1991). Sugar (glucose, xylose, and arabinose), xylitol, and ethanol concentrations were determined by high-performance liquid chromatography (HPLC) (Agilent Technologies, HP1260, United States) using a Hi-Plex H column (Agilent Technologies, United States) and refractive index detector (RID) with similar mobile phase and running conditions as in the work of Tangtua et al. (2013). The concentrations of inhibitors, namely, furfural, HMF, and acetic acid were analyzed by HPLC with a 250 × 4.6 mm 5 μm ZORBAX Eclipse XDB-C18 column at 30°C and a diode array detector (DAD) at 210 nm (acetic acid) as well as 263 nm (HMF and furfural) with 5 μL injection volume. A gradient of acetonitrile:5 mM sulfuric acid was used as an eluent starting with 0:100 at 0 min, 10:90 at 6 min, 30:70 at 12 min, 70:30 at 18 min, 98:2 at 24 min, and 100:0 at 30 min and held until 35 min with a flow of 0.8 mL/min (modified from the work of Ohra-aho et al. (2021), and Dhanani et al. (2014), and Pereira et al. (2010)). Dried biomass concentration was determined as previously described by Leksawasdi et al. (2004). The morphological structures of SCB, RS, and CC in different stages, namely, original pulverized powder, solid portion after the pretreatment step, and residual solid portion after the enzymatic hydrolysis step, were compared using a scanning electron microscope (SEM) (JSM-IT300, JEOL, Japan) with photomicrographs taken at ×200 magnification. Kinetic parameters of yields (Y), specific growth rate (μ), specific substrate consumption or product formation rates (q), and productivity (Q) such as Y_Xy/Xyl_, Y_Et/TotS_, Y_X/TotS_, μ_max_, q_TotS,max_, q_Xy,max_, q_Et,max_, Q_Xy,max_, and Q_Et,max_ were calculated based on methods described previously (Mahakuntha et al., 2021; Nunta et al., 2023). The theoretical mass yields of xylitol and ethanol of 0.912 g xylitol/g xylose (Tamburini et al., 2013) and 0.511 g ethanol/g xylose or glucose (Okamoto et al., 2014; Li Y. et al., 2019) were used for comparison with experimental values and expressed in relative percentages. The full names of subscript abbreviations are given in Nomenclature. Bz, Pyr, PAC, and by-products (acetaldehyde, acetoin, benzyl alcohol, and benzoic acid) were quantified by HPLC at 283 nm using the Altima^TM^ C8 column and DAD (Khemacheewakul et al., 2018). Volumetric PDC carboligase activity and the respective definition of activity were also determined with a similar procedure and definition as mentioned previously (Rosche et al., 2002) for collected frozen–thawed whole cells at 240 h for subsequent biotransformation experiment. The specific PDC activity per gram of frozen–thawed whole cells was also calculated based on wet whole cells being weighed and solubilized in a known volume of carboligase buffer. PAC molarity yields and assessment of close molarity balances with respect to Pyr and Bz were based on the well-established methods published elsewhere (Kumar et al., 2023). Costing analyses relevant to PAC production in both single-phase and two-phase emulsion systems utilizing frozen–thawed whole cells were performed with the well-established strategy published previously by our group (Leksawasdi et al., 2021; Kumar et al., 2023).

### 2.13 Hypothesis testing

Mean values (MVs) and respective standard errors (SEs) were evaluated from experimental data using at least three replicates and represented as MV ± SE. The statistically significant difference (*p* ≤ 0.05) of results was analyzed using SPSS for Windows 22.0 (SPSS, United States). ANOVA in this case was carried out using Duncan’s multiple-range test with the similar *p*-value probability. Two adjacent mean values (MV_1_ and MV_2_) between subsequent time courses which were not statistically significant different (*p* ≤ 0.05) were shown in the range with the highest SE among the two or (MV_1_–MV_2_) ± SE_max_.

## 3 Results

### 3.1 Optimization of the pretreatment step using the RSM via the CCD

**FIGURE 1:**
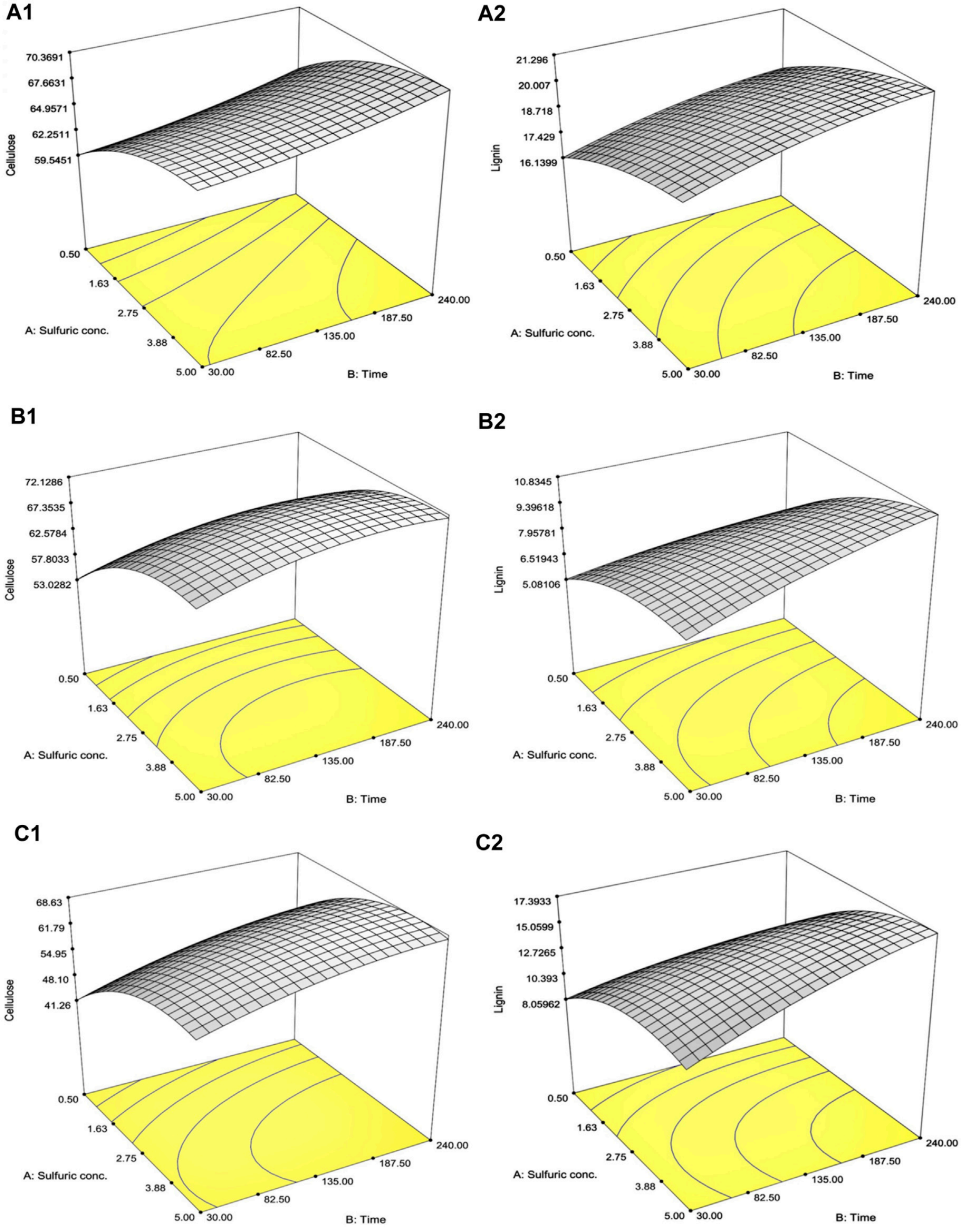
Response surface plot of the interaction effects between diluted [sulfuric acid] and treatment time on **(A)** SCB, **(B)** RS, and **(C)** CC on the optimization of the remaining **(1)** cellulose and **(2)** lignin contents in the solid portion.

A total of 13 experiments designed by the CCD for SCB, RS, and CC with diluted [sulfuric acid] (0.5%–5.0% w/v) and treatment time (30–240 min) as well as the results of the remaining cellulose and lignin after pretreatment are tabulated in Table 2. The effect of increasing [sulfuric acid] and treatment time was directly proportional to remnant cellulose and lignin contents in solid residue reaching the highest responses as indicated in Run number 4 with the highest diluted [sulfuric acid] and treatment time. This was compared to the lowest lignin content in Run number 1 with the lowest diluted [sulfuric acid] and treatment time. A series of regressed quadratic equations for SCB, RS, and CC which correlated diluted [sulfuric acid] and treatment time to the remaining cellulose (Eqs 3.1–3.3) and lignin (Eqs 4.1–4.3) contents in the solid residue were generated by the CCD based on actual factors. The assessment of each term in the regressed equation by the *F*-test and ANOVA revealed that the combined equation or model term significant for predicting responses for SCB, RS, and CC were all statistically significant (*p* ≤ 0.05) with the response surface plot as shown in Figure 1. In fact, *R* ^2^ and Adj-*R* ^2^, which are also included in Table 2, implied high correlation (≥0.9) of diluted [sulfuric acid] and treatment time to the remaining cellulose and lignin contents in the solid residue. Furthermore, CV values (Table 2) which were all lower than 10% also indicated better precision and reliability of the regressed equations in predicting experimental data. Supplementary Tables S1–S3 list other relevant statistical parameters for SCB, RS, and CC pretreatment optimization. The models were then used to predict the optimal cellulose and lignin contents in the solid residue after the pretreatment step. The targeted responses would be the optimum balanced values between relatively high cellulose with sufficiently low lignin contents. These optimal pretreatment conditions (diluted [sulfuric acid], treatment time) as suggested by the models were (3.36% w/v, 113 min), (3.77% w/v, 109 min), and (3.89% w/v, 112 min), for SCB, RS, and CC, respectively. The corresponding predicted responses (cellulose content, lignin content) in the solid residue were (66.7, 19.7% w/w), (70.1, 8.84% w/w), and (64.5, 14.5% w/w), for SCB, RS, and CC, respectively. Table 3 shows the validation results of these models by independent verification experiments producing the actual responses (cellulose content, lignin content) in the solid residue of (68.0 ± 0.3, 19.5% ± 0.1% w/w), (68.2 ± 1.1, 8.54% ± 0.09% w/w), and (61.6 ± 0.8, 16.0% ± 0.4% w/w), for SCB, RS, and CC, respectively. The paired comparison of predicted and actual responses clearly elucidated the acceptable relative errors of less than 10%.

$${SCB\;cellulose\;content\;(Y}_{1,SCB})=+57.63593+3.44509X_{1}+7.92567\times 10^{-3}X_{2}-0.33455X_{1}^{2}+4.38150\times 10^{-5}X_{2}^{2}-4.62041\times 10^{-4}X_{1}X_{2},$$ (3.1)

$${RS\;cellulose\;content\;(Y}_{1,RS})={+47.40221+7.60160X}_{1}+0.073388X_{2}-0.83442X_{1}^{2}-1.99685\times 10^{-4}X_{2}^{2}+7.89559\times 10^{-4}X_{1}X_{2},$$ (3.2)

$${CC\;cellulose\;content\;(Y}_{1,CC})={+33.30636+10.49540X}_{1}+0.10705X_{2}-1.20358X_{1}^{2}-1.88237\times 10^{-4}X_{2}^{2}-2.36237\times 10^{-3}X_{1}X_{2},$$ (3.3)

$${SCB\;lignin\;content\;(Y}_{2,SCB})=+15.05065+1.14863X_{1}+0.018857X_{2}-0.089758X_{1}^{2}-3.35452\times 10^{-5}X_{2}^{2}+1.27350\times 10^{-4}X_{1}X_{2},$$ (4.1)

$$RS\;lignin\;content\;\left(Y_{2,RS}\right)=+4.07837+1.41683X_{1}+0.010830X_{2}-0.16762X_{1}^{2}-2.51179\times 10^{-5}X_{2}^{2}+2.25835\times 10^{-3}X_{1}X_{2},$$ (4.2)

$${CC\;lignin\;content\;(Y}_{2,CC})=+6.13372+2.62440X_{1}+0.023158X_{2}-0.37001X_{1}^{2}-6.33511\times 10^{-5}X_{2}^{2}+4.56576\times 10^{-3}X_{1}X_{2}.$$ (4.3)

**FIGURE 2:**
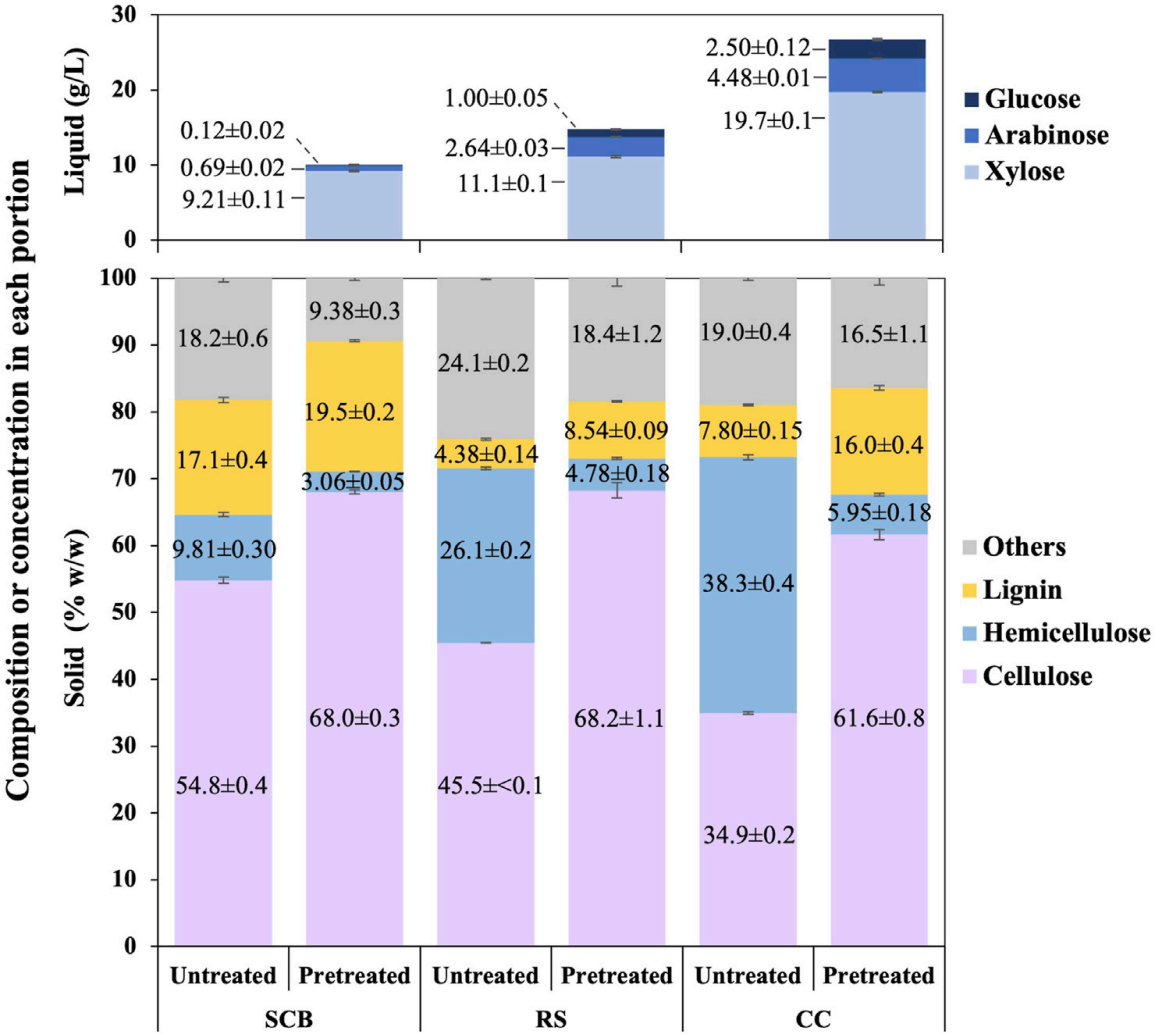
Composition of cellulose, hemicellulose, lignin, and others in the solid portion as well as [xylose], [arabinose], and [glucose] in the liquid portion after the pretreatment step of SCB, RS, and CC.

The employed optimized conditions resulted in compositions of cellulose, hemicellulose, and lignin in the solid portion and concentrations of xylose, arabinose, and glucose in the liquid portion after the pretreatment step of SCB, RS, and CC, as shown in Figure 2. The comparison of solid compositions was also being made to each untreated lignocellulosic biomass. Under these optimal conditions, the composition of cellulose was increased by 13.2 ± 0.6, 22.8 ± 1.1, and 26.7% ± 0.8% w/w in SCB, RS, and CC, respectively. Lignin compositions were also enhanced by 2.41 ± 0.41, 4.17 ± 0.17, and 8.17% ± 0.38% w/w for SCB, RS, and CC, respectively. There appeared a correlation of hemicellulose solubilization in the solid portion with the presence of xylose, arabinose, and glucose in the liquid portion after the pretreatment step. Some hemicellulose composition in the untreated lignocellulosic biomass might have disintegrated and resulted in the decrease in hemicellulose by 6.74 ± 0.30, 21.3 ± 0.3, and 32.3% ± 0.4% w/w in SCB, RS, and CC, respectively. The soluble pentose and hexose monosaccharides in the liquid portion could be detected as evident in Figure 2. The mass balance of disappearing hemicellulose in the solid portion after the pretreatment step suggested that the degraded hemicellulose might also exist in a more complex form of saccharides rather than solely monosaccharides such as disaccharides or oligosaccharides (de Jong and Gosselink, 2014). The remnant liquid portion for SCB, RS, and CC was 74, 81, and 80 mL, respectively. The overall monosaccharide concentration in the liquid portion as shown in Figure 2 could be calculated back to hemicellulosic compositions in the untreated SCB, RS, and CC of 6.66 ± 0.13, 10.7 ± 0.1, and 19.2% ± 0.1% w/w, respectively. The initial hemicellulosic compositions in SCB, RS, and CC were converted to monosaccharides in the liquid portion by 67.9% ± 2.1%, 41.1% ± 0.3%, and 50.2% ± 0.5%, respectively. The highest sugar conversions were obtained from CC with 144 ± 1, 32.7 ± 0.1, 18.3 ± 0.8, and 195 ± 1 mg/g pulverized powder for xylose, arabinose, glucose, and total sugars, respectively, followed by the conversion of RS and SCB with (xylose, arabinose, glucose, and total sugars) as (81.1 ± 1.0, 19.2 ± 0.2, 7.32 ± 0.39, and 108 ± 1 mg/g pulverized powder) and (67.3 ± 0.8, 5.07 ± 0.17, 0.90 ± 0.16 mg/g pulverized powder), respectively.

### 3.2 Optimization of the enzymatic hydrolysis step using the RSM via the CCD

**FIGURE 3:**
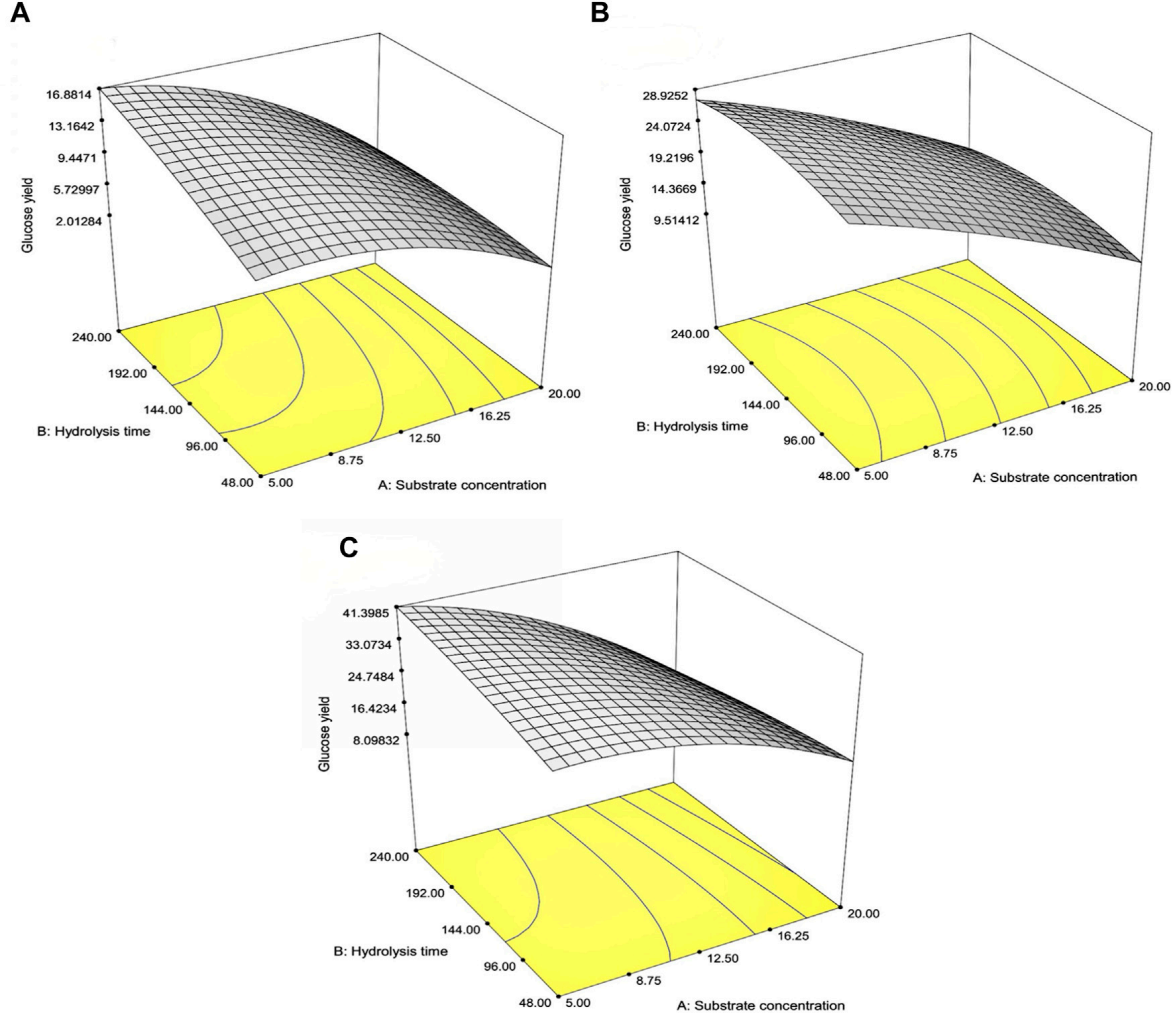
Response surface plot of the interaction effects between substrate concentration and hydrolysis time on the glucose percentage yields for **(A)** SCB, **(B)** RS, and **(C)** CC in the optimization of the enzymatic hydrolysis step.

Table 4 indicates the 13 experimental designs by the CCD for the pretreated SCB, RS, and CC solids obtained from the prior optimization with [pretreated solids] (5%–20% w/v) and hydrolysis time (48–240 h) as well as the result of glucose yield. The maxima glucose yields for SCB and CC were obtained at Run number 3, of which the lowest [pretreated solids] and the highest hydrolysis time were used. This was in contrast to Run number 5 where the maximum glucose yield of RS was attained with the lowest [pretreated RS] and hydrolysis time of 144 h. Further increase in hydrolysis time for RS did not result in the improved glucose yield, possibly due to the relatively low lignin content for this lignocellulosic biomass compared to the others. As lignin is a well-known compound which exerts some degree of enzymatic inhibitory effect, the extended enzymatic hydrolysis times are, thus, required for the digestion of pretreated SCB and CC to achieve the maximum glucose yield as RS. A similar form of regressed quadratic equations described previously for pretreatment step optimization was, thus, employed for the correlation of [pretreated solids] and enzymatic hydrolysis time to glucose yield (Eqs 5.1–5.3) in the enzymatic hydrolysis step based on actual factors. The *F*-test and ANOVA clearly showed a statistically significant (*p* ≤ 0.05) response surface plot (Figure 3) with relatively high correlation (>0.9) of [pretreated solids] and enzymatic hydrolysis time to glucose yield as evident from *R* ^2^ and Adj-*R* ^2^ (Table 4). CV values were in the range of 6.81%–8.82% which was lower than 10%, indicating improved precision and reliability of the regressed equations in predicting experimental data. Supplementary Tables S6–S8 tabulate other relevant statistical parameters for SCB, RS, and CC enzymatic hydrolysis optimization. The regressed quadratic equations obtained previously were used to optimize glucose yield after the enzymatic hydrolysis step with relatively high [pretreated solids] as much as possible to increase substrate loading and minimize hydrolysis time for productivity enhancement. The elucidated optimal enzymatic hydrolysis conditions ([pretreated solids], hydrolysis time) from the model were (12.1% w/v, 93 h), (10.9% w/v, 61 h), and (12.0% w/v, 90 h) for SCB, RS, and CC, respectively. The predicted glucose yields in liquid portion after the enzymatic hydrolysis step for SCB, RS, and CC were 11.2% w/w, 21.6% w/w, and 30.1% w/w, respectively. The validated glucose yields obtained from the specified optimal enzymatic hydrolysis conditions were 11.7% ± 0.2% w/w, 23.5 ± < 0.1% w/w, and 32.7% ± 0.4% w/w, respectively. The subsequent determination of relative errors between these predicted and validated values showed that they were all lower than 10% (Table 5). Further analyses of the liquid portion after the enzymatic hydrolysis step of SCB, RS, and CC for [monosaccharides] indicated [glucose] of 21.3 ± 0.3 g/L, 34.6 ± 0.1 g/L, and 51.5 ± 0.6 g/L with the slight [xylose] of 5.92 ± 0.09 g/L, 7.34 ± 0.28 g/L, and 10.6 ± 0.3 g/L, respectively. The highest sugar conversions were obtained from CC at which glucose, xylose, and total sugars of 149 ± 3, 33.5 ± 1.4, and 182 ± 4 mg/g pretreated solid were recorded, respectively. The glucose, xylose, and total sugar conversion of RS and SCB were (118 ± <1, 29.7 ± 1.5, and 148 ± 1 mg/g pretreated solid) and (88.3 ± 1.2, 24.5 ± 0.4, and 113 ± 2 mg/g pretreated solid), respectively.

$$SCB\;glucose\;yield\;\left(Y_{a,SCB}\right)=+3.87492+0.89196X_{a}+0.066674X_{b}-0.051233X_{a}^{2}-5.78177\times 10^{-5}X_{b}^{2}-2.37007\times 10^{-3}X_{a}X_{b},$$ (5.1)

$$RS\;glucose\;yield\;\left(Y_{a,RS}\right)=+28.17722-0.66821X_{a}+0.060399X_{b}-0.019257X_{a}^{2}-1.98187\times 10^{-4}X_{b}^{2}-4.04705\times 10^{-5}X_{a}X_{b},$$ (5.2)

$$CC\;glucose\;yield\;\left(Y_{a,CC}\right)=+23.05893+1.50643X_{a}+0.10618X_{b}-0.095189X_{a}^{2}-9.65649\times 10^{-5}X_{b}^{2}-5.61129\times 10^{-3}X_{a}X_{b}.$$ (5.3)

**TABLE 4:** Experimental design and CCD responses for the optimization of the enzymatic hydrolysis step.

Run number	Variables (*X*)		Responses (*Y*)		
			SCB	RS	CC
	*X*_a_	*X*_b_	*Y*_a,SCB_	*Y*_a,RS_	*Y*_a,CC_
1	5.00	48	10.2	28.1	32.0
2	20.0	48	2.77	10.4	16.4
3	5.00	240	**16.7**	26.5	**39.8**
4	20.0	240	2.42	***8.73***	***8.00***
5	5.00	144	13.3	**28.5**	38.8
6	20.0	144	***2.14***	12.0	10.6
7	12.5	48	7.25	17.0	25.6
8	12.5	240	12.9	21.9	31.8
9^a^	12.5	144	11.9	19.6	29.6
10^a^	12.5	144	10.8	21.7	31.1
11^a^	12.5	144	11.2	21.3	32.1
12^a^	12.5	144	11.1	21.8	29.5
13^a^	12.5	144	11.9	22.3	29.0
*p*-value			<0.0001	<0.0001	<0.0001
*R* ^2^			0.98	0.96	0.98
Adj-*R* ^2^			0.96	0.93	0.96
CV (%)			8.82	8.18	6.81

*X*_a_ = [pretreated solids] (% w/v), *X*_b_ = hydrolysis time (h), *Y*_a_ = glucose yield (% w/w). Bolded numbers indicate the highest values. Bolded, and italicized numbers indicate the lowest values.

^a^ Quintuplicate were applied at the center point.

**TABLE 5:** Model validation of the optimal enzymatic hydrolysis conditions with the corresponding predicted, actual, and relative errors of glucose percentage yields for SCB, RS, and CC.

Pretreated lignocellulosic material	[Pretreated solid] (% w/v)	Time (h)	Predicted values (% w/w)	Actual values (% w/w)	Relative errors (%)
SCB	12.1	93	11.2	11.7 ± 0.2	4.68
RS	10.9	61	21.6	23.5 ± <0.1	9.04
CC	12.0	90	30.1	32.7 ± 0.4	8.73

**FIGURE 4:**
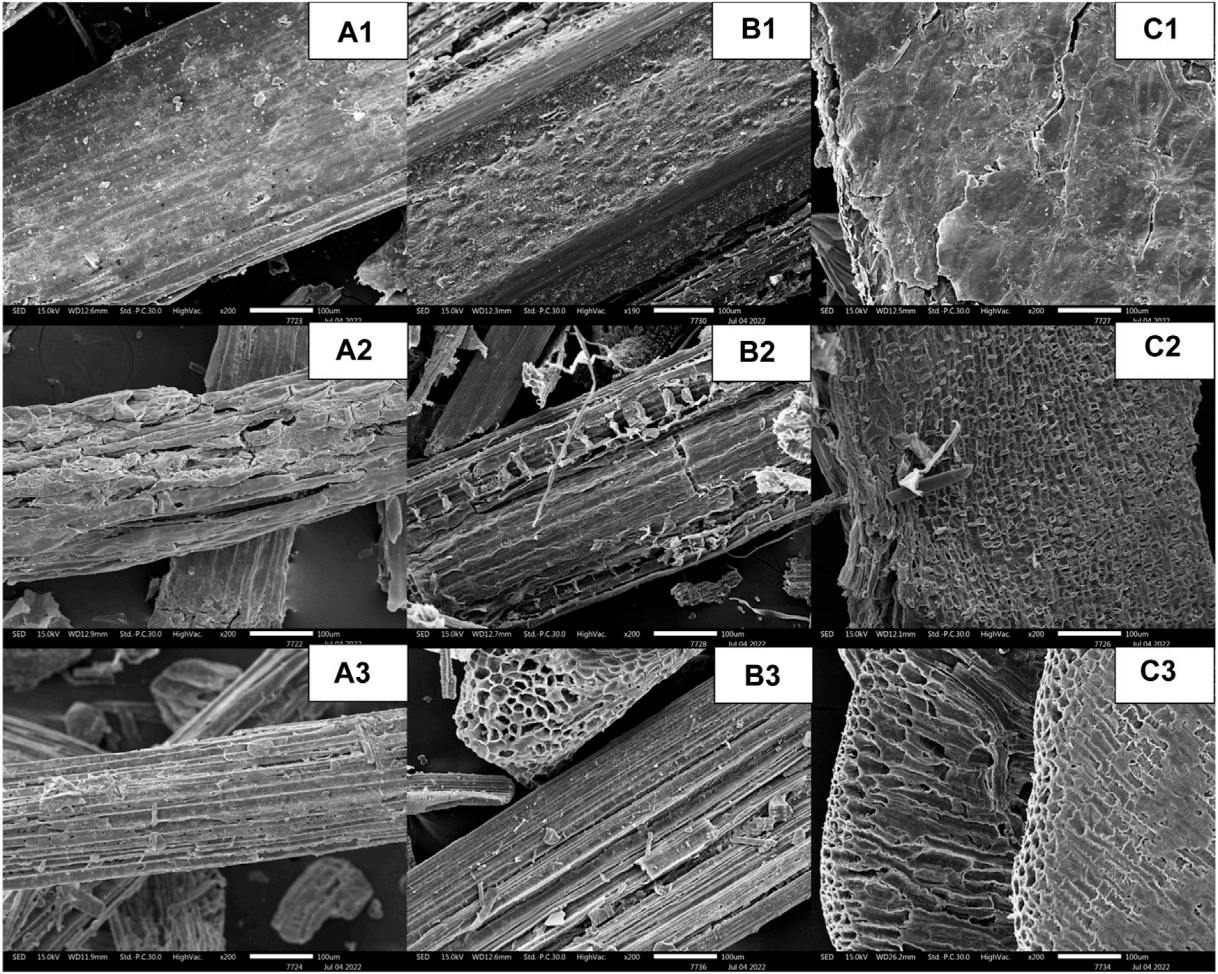
SEM images at ×200 magnification of **(A)** SCB, **(B)** RS, and **(C)** CC in three stages, namely, **(1)** original pulverized powder, **(2)** the solid portion after the pretreatment step, and **(3)** the residual solid portion after the enzyme hydrolysis step.

The SEM images of the original pulverized powder, solid portion after the pretreatment step, and residual solid portion after the enzyme hydrolysis step were taken at ×200 magnification (Figure 4). The micrographs of untreated SCB, RS, and CC (Figures 4A1–C1) indicated the even and smooth flat surfaces of cell walls. After diluted sulfuric acid pretreatment, the effect of acidic breakage at the susceptible glycosidic linkages between hemicellulose and cellulose could be clearly seen on the surfaces. As the hemicellulose was dissolved, the microfibril structures appeared to open up with increased porosity and the presence of more crystalline cellulose structures (Figures 4A2–C2). Some of the lignin structures also appeared in the pretreated RS as shown in Figure 4B2. The enhanced crystalline structure of cellulose fibrils was also evident after the enzymatic hydrolysis step.

### 3.3 Production of xylitol and ethanol

CC was the lignocellulosic material of choice for xylitol and ethanol production, as evident from prior optimization experiments for pretreatment and enzymatic hydrolysis steps. The [xylose] of 19.7 ± 0.1 g/L in the solid portion of the pretreatment step and glucose yield (32.7% ± 0.4% w/w) as well as [glucose] (51.5 ± 0.6 g/L) in the liquid portion enzymatic hydrolysis step of CC were all statistically significantly highest (*p* ≤ 0.05) among other lignocellulosic materials.

Initially, for xylitol production, [glucose], [xylose], [arabinose], and [total sugars] were evident at 8.52 ± 0.13, 53.0 ± 1.2, 16.3 ± 0.6, and 77.8 ± 1.8 g/L, respectively. From Table 6, the statistically significant maximum (*p* ≤ 0.05) [xylitol] was (28.4–29.1) ± 0.3 g/L with a Y_Xy/Xyl_ of (0.58–0.60) ± 0.01 g_Xy_/g_Xyl_ which corresponded to (64–66) ± 1% of the xylitol theoretical yield obtained at 72–96 h after cultivation. At the same time interval, a relatively low [ethanol] of (3.89–5.98) ± 0.30 g/L was also attained with the Y_Et/TotS_ of (0.056–0.093) ± 0.003 g_Et_/g_TotS_ or (11–18) ± 1% of the ethanol theoretical yield. A [dried biomass] of (6.67–10.8) ± 0.32 g/L which corresponded to a Y_X/TotS_ of (0.10–0.16) ± 0.01 g_X_/g_TotS_ was obtained. In the current system, the statistically significant highest (*p* ≤ 0.05) level of [ethanol] being produced was (5.87–5.98) ± 0.30 g/L with the corresponding Y_Et/TotS_ of (0.093–0.10) ± 0.003 g_Et_/g_TotS_ observed at 48–72 h. This was compared to the statistically significant highest (*p* ≤ 0.05) level of [dried biomass] generated at (17.7–18.4) ± 0.5 g/L and a Y_X/TotS_ of (0.25–0.26) ± 0.01 g_X_/g_TotS_ at 192–240 h.

**TABLE 6:** Xylitol and ethanol and dried biomass production with the relative kinetic parameters using xylose- and glucose-rich hydrolysates without the detoxification step as carbon sources.

Concentration and kinetic parameter	Xylose-rich hydrolysate in an orbital shaking with a microaerobic condition system	Glucose-rich hydrolysate in an orbital shaking with a partially anaerobic condition system
Optimal cultivation time (h)	72–96	144–240
[Xylitol] (g/L)	**(28.4–29.1)** ^**A**^ **± 0.3**	(5.13–5.59)^B^ ± 0.38
[Ethanol] (g/L)	(3.89–5.98)^B^ ± 0.30	**(46.9–48.0)** ^**A**^ **± 0.4**
[Dried biomass] (g/L)	**(6.67–10.8)** ^**A**^ **± 0.32**	(0.51–1.48)^B^ ± 0.09
Y_Xy/Xyl_ (g_Xy_/g_Xyl_)	**(0.58–0.60)** ^**A**^ **± 0.01**	(0.27–0.39)^B^ ± 0.03
Y_Et/TotS_ (g_Et_/g_TotS_)	(0.06–0.09)^B^ ± < 0.01	**(0.43–0.45)** ^**A**^ **± 0.01**
Y_X/TotS_ (g_X_/g_TotS_)	**(0.10–0.16)** ^**A**^ **± 0.01**	(0.004–0.01)^B^ ± 0.01
μ_max_ (h^−1^)	**0.016** ^**A**^ **± 0.001**	**0.014** ^**A**^ **± 0.001**
q_TotS,max_ (g_TotS_/g_X_/h)	**−0.79** ^**A**^ **± 0.05**	−0.92^B^ ± 0.04
q_Xy,max_ (g_Xy_/g_X_/h)	**0.30** ^**A**^ **± 0.02**	0.10^B^ ± < 0.01
q_Et,max_ (g_Et_/g_X_/h)	0.17^B^ ± < 0.01	**0.45** ^**A**^ **± 0.02**
Q_Xy,max_ (g_Xy_/L/h)	**0.40** ^**A**^ **± < 0.01**	0.039^B^ ± 0.002
Q_Et,max_ (g_Et_/L/h)	0.083^B^ ± 0.004	**0.33** ^**A**^ **± < 0.01**

Xy = xylitol; Et = ethanol; X = dried biomass; Xyl = xylose; TotS = total sugars. Numbers with the same superscript capital alphabet indicate no significant difference (*p* > 0.05) for the comparison of the same row.

Bold values indicated the statistical significantly highest in the same row.

The initial [glucose], [xylose], and [total sugars] were 105 ± 2, 29.3 ± 0.8, and 135 ± 3 g/L, respectively, for ethanol production. The statistically significant maximum (*p* ≤ 0.05) [ethanol] was (46.9–48.0) ± 0.4 g/L with a Y_Et/TotS_ of (0.43–0.45) ± 0.01 g_Et_/g_TotS_ which corresponded to (84–88) ± 2% of the ethanol theoretical yield based on total sugar utilization achieved at 144–240 h. At this time duration, the [xylitol] was also produced with the statistically significant highest (*p* ≤ 0.05) values of (5.17–5.59) ± 0.38 g/L with the corresponding Y_Xy/Xyl_ of (0.31–0.39) ± 0.04 g_Xy_/g_Xyl_ or (37–42) ± 3% of the xylitol theoretical yield. [Dried biomass] of (0.51–1.48) ± 0.12 g/L and the corresponding Y_X/TotS_ of less than 0.015 g_X_/g_TotS_ were detected. The statistically significant highest (*p* ≤ 0.05) level of [dried biomass] generated at 4.39 ± 0.18 g/L and Y_X/TotS_ of 0.05 ± < 0.01 g_X_/g_TotS_ was observed at 96 h.

The sugar mixture was both xylose-rich and glucose-rich from the respective pretreatment, and enzymatic hydrolysis could also be used for simultaneous xylitol and ethanol co-production with *Candida* spp. Evidently, the relatively high ethanol yield was observed with lower Y_Xy/Xyl_ (around 0.2 g_Xy_/g_Xyl_) which may indicate that suitable conditions for xylitol and ethanol production are dissimilar. Xylitol should be produced under limited-aerobic conditions while ethanol should be produced under anaerobic conditions. Such findings were also observed in various studies (Du et al., 2020; Raj and Krishnan, 2020; Hor et al., 2023).

### 3.4 The presence of inhibitory compounds from the pretreatment to cultivation steps

The formation and degradation of three possible inhibitors, namely, HMF, furfural, and acetic acid, were observed in all steps of pretreatment, evaporation, and cultivation. Table 7 reveals that CC possessed the statistically significant highest (*p* ≤ 0.05) concentration of all three inhibitors (319 ± 15 mg/L for HMF, 122 ± < 1 mg/L for furfural, 3.11 ± 0.14 g/L for acetic acid). This was strongly correlated to the relatively high monosaccharide conversion (Sajid et al., 2021) when compared with SCB (1.21 ± 0.26 mg/L, 43.1 ± 2.0 mg/L, and 1.07 ± 0.02 g/L) and RS (50.5 ± 1.0 mg/L, 58.5 ± 1.4 mg/L, and 1.11 ± 0.04 g/L) during the pretreatment step.

**TABLE 7:** Furfural, HMF, and acetic acid concentrations in original acid hydrolysate, before xylitol production, and after xylitol production.

Xylose-rich hydrolysate	Lignocellulosic material	[Furfural] (mg/L)	[HMF] (mg/L)	[Acetic acid] (g/L)
Original hydrolysate	SCB	43.1^Bc^ ± 2.0	1.21^Bd^ ± 0.26	**1.07** ^**Ac**^ **± 0.02**
	RS	58.5^Bb^ ± 1.4	50.5^Bc^ ± 1.0	**1.11** ^**Ac**^ **± 0.04**
	CC	**122** ^**Ca**^ **± < 1**	319^Bb^ ± 15	**3.11** ^**Ab**^ **± 0.14**
Before xylitol production	CC	33.8^Cd^ ± 1.3	**484** ^**Ba**^ **± 2**	**4.28** ^**Aa**^ **± 0.12**
After xylitol production	CC	9.23^Be^ ± 0.30	13.0^Bd^ ± 0.5	**0.43** ^**Ad**^ **± 0.07**

Numbers with the same superscript capital and small alphabets indicate no significant difference (*p* > 0.05) for the comparison of the same row and column, respectively.

[HMF] and [acetic acid] after vacuum evaporation and pH adjustment steps prior to cultivation were 484 ± 2 mg/L and 4.28 ± 0.12 g/L, respectively, which corresponded to the statistically significant increase (*p* ≤ 0.05) by 51.7% ± 4.7% and 37.6% ± 5.9% of those in the original hydrolysate, whereas [furfural] was statistically significantly decreased (*p* ≤ 0.05) by 72.3% ± 1.1% to only 33.8 ± 1.3 mg/L.

After 72 h cultivation during xylitol production, the wild type of *C. magnoliae* TISTR 5664 could statistically significantly degrade (*p* ≤ 0.05) these inhibitors (HMF, acetic acid, and furfural) by 97.3% ± 0.4% (final value of 13.0 ± 0.5 mg/L), 90.0% ± 3.2% (final value of 0.43 ± 0.07 g/L), and 76.2% ± 3.4% (final value of 9.23 ± 0.30 mg/L), respectively. In fact, acetic acid was totally consumed after 96 h.

### 3.5 PAC biotransformation in the two-phase emulsion system

The specific PDC activity of 4.75 ± 0.10 and 2.50 ± 0.05 U/g frozen–thawed whole cells from xylitol and ethanol production systems, respectively, was elucidated before use. The [PAC] of 44.0 ± 1.7, 96.2 ± 3.2, and 59.7 ± 0.2 mM were achieved in aqueous, organic, and overall phases, respectively, at 8 h reaction time. The overall [PAC] was improved by 2-fold compared to the value previously reported by Khemacheewakul et al. (2018) who utilized Pi buffer as a single-phase emulsion system. The comparison of PAC production without pH control between single-phase and two-phase emulsion systems is tabulated in Table 8. This result showed a similar magnitude of [PAC] production in overall phases when compared with 62.3 mM from the latest published report using *C. tropicalis* whole cells obtained from ethanol production (Nunta et al., 2023). The PAC molar yields on Pyr and Bz were 0.71 ± 0.03 mol PAC/mol Pyr and 0.95 ± 0.04 mol PAC/mol Bz, respectively. The Pyr and Bz molarity balances were 99% ± 6% and 88% ± 2%, respectively.

**TABLE 8:** Comparison of PAC production without pH control in **(A)** the single-phase emulsion system and organic/buffer two-phase emulsion system, **(B**–**C)** octanol/2.5 M MOPS, and **(D)** vegetable oil/1 M Pi.

Variable	Single-phase emulsion system	Organic/buffer two-phase emulsion system		
	1 M Pi	Octanol/2.5 M MOPS		Vegetable oil/1 M Pi (current study)
V_org_:V_aq_	0:1 (A)^a^	1:1 (B)^b^	0.43:1 (C)^c^	0.43:1 (D)
Process time (h)	3	49	48	8
Temperature (°C)	10	6	4	10
PAC_org_ (mM)	-	939	1,218	96.2 ± 3.2
PAC_aq_ (mM)	28.6 ± 2.3	120	178	44.0 ± 1.7
PAC_overall_ (mM)	28.6 ± 2.3	529	491	59.7 ± 0.2
Initial act (U/mL)	2.47 ± 0.07	8.5	2.8	1.53 ± 0.04
Residual act (%)	19.5 ± 3.0	23	60%–70%^d^	78.0 ± 3.7
S_p,PAC_ (mg/U_ICA_)	1.74 ± 0.15	19	23.5	5.88 ± 0.15
Q_PAC_ (mM/h)	9.53 ± 0.02	10.8	10.2	7.46 ± 0.02
Y_PAC/Pyr_ (mol_PAC_/mol_Pyr_)	0.71 ± 0.06	0.73	0.95	0.71 ± 0.03
Y_PAC/Bz_ (mol_PAC/_mol_Bz_)	0.95 ± 0.08	0.90	0.99	0.95 ± 0.04
Pyr balance (%)	N/A	89	107	99 ± 6
Bz balance (%)	N/A	90	100	88 ± 2

^a^ Khemacheewakul et al. (2018).

^b^ Sandford et al. (2005).

^c^ Gunawan et al. (2008).

^d^ Graphical estimation from the work of Gunawan et al. (2008).

There was no acetaldehyde and acetoin being formed during the 8 h reaction time course as evident from the final Pyr molarity balance which was almost 100%. Benzyl alcohol was also not detected, possibly due to the inactivity of alcohol dehydrogenase (ADH) in the frozen–thawed whole cells (Khemacheewakul et al., 2021). Benzoic acid was the sole by-product being generated in a minute amount of 1.75 ± 0.09 mM. The relatively low Bz molarity balance might reflect some losses (12%), which confirms the higher Bz volatility compared to Pyr (Kumar et al., 2023).

## 4 Discussion

The implementation of high temperature and high pressure could affect the sugar conversion yields in the pretreatment step. These results indicated slightly low sugar conversions when compared to the values previously reported by Sumphanwanich et al. (2008) who employed 121°C with diluted sulfuric acid and an LSR of 10:1 (v/w) for 1 h reaction time in pretreatment. The optimal diluted [sulfuric acid] of 188 mM (1.84% w/v) was achieved for SCB and CC and 282 mM (2.77% w/v) for RS. The reducing sugar conversion showed a higher release up to 402 ± 1.2, 255 ± 4.9, and 473 ± 2.5 mg/g dried solid for CC, SCB, and RS, respectively, with the statistically significant highest (*p* ≤ 0.05) xylose released of 206 ± 6.1 mg/g dried solid obtained from CC, followed by SCB (120 ± 0.5 mg/g dried solid) and RS (119 ± 0.4 mg/g dried solid), respectively. The diluted sulfuric acid pretreatment step with less than 100°C might seem to be more suitable for industrial applications due to lower energy cost. The reported glucose yields were slightly lower when compared to high temperature ranges (100°C–250°C) (Baruah et al., 2018). Sumphanwanich et al. (2008) reported the highest reducing sugars released after diluted sulfuric acid pretreatment at 121°C followed by enzymatic hydrolysis at 50°C and pH 4.5 for 48 h using 5% w/v of pretreated solid. The corresponding values for CC, SCB, and RS were 694 ± 2.6 mg/g, 520 ± 1.6 mg/g, and 466 ± 4.2 mg/g, respectively. The appending of the alkaline pretreatment step could also be applied to remove the remaining lignin content after hemicellulose was solubilized in acid hydrolysate (Tan et al., 2021; Antunes et al., 2023) which, in turn, resulted in the enhanced glucose conversion yield in the enzymatic hydrolysis step.

Evidently, the remaining lignin content in the pretreated solids could inhibit enzyme accessibility, resulting in low glucose yield. It is possible that the abundant S-type lignin of up to 60% w/w in the pretreated SCB with a relatively high molecular weight of 210.23 g/mol and predominant *β*-ether linkage bestows upon this type of lignocellulosic biomass with high degree of resistivity when subjected to breakdown by various acids and alkaline solutions at physiological temperature. On the other hand, RS contains a rather rich G type (68% w/w) with a molecular weight of 180.20 g/mol, while the CC structure is mainly associated with the H type (55% w/w) with the lowest molecular weight of 150.17 g/mol (del Río et al., 2015; Rabemanolontsoa and Saka, 2013; Smith et al., 2022; Takada et al., 2018). Thus, the pretreated SCB with the highest lignin content (19.5% ± 0.2% w/w) and highly resistant structure had the lowest glucose conversion yield after passing through the enzymatic hydrolysis step when compared with the other two counterparts.

The results of xylitol and ethanol production using the hydrolysates obtained from the optimal pretreatment and enzymatic hydrolysis steps showed that *C. magnoliae* TISTR 5664 in this study could consume up to 97% of available xylose resulting in the similar reported Y_Xy/Xyl_ of 0.57 ± 0.05 g_Xy_/g_Xyl_ using the same strain in 25 g/L xylose medium (Hor et al., 2023). Nevertheless, the xylitol yield in the current study was higher compared to 0.452 g_Xy_/g_Xyl_ obtained from *C. magnoliae* TISTR 5663 in a non-detoxified SCB hydrolysate (Wannawilai and Sirisansaneeyakul, 2015). Another study of *C. magnoliae* by Arrizon et al. (2012) reported a relatively lower xylitol yield of 0.24 ± 0.01 g_Xy_/g_Xyl_ using SCB xylose-rich hydrolysate. In fact, the xylitol yield could be improved by optimizing the agitation intensity and/or air flow rate as well as employing a two-stage aeration rate using high aeration in the first stage and a subsequent decrease in in the second stage in a fermenter system (Raj and Krishnan, 2020; Hor et al., 2023). The Y_Xy/Xyl_ of 0.83 ± 0.08 g_Xy_/g_Xyl_ was achieved from this two-stage aeration fermentation (Hor et al., 2023). In commercial ethanol production, *S*. *cerevisiae* was the obvious choice for the most favorable yeast employed. The ethanol production reported by Arrizon et al. (2012) showed a similarly high ethanol production yield of 0.44 ± 0.02 g_Et_/g_Glu_, which corresponded to 85.4% ± 2.4% of the theoretical yield using non-detoxified SBC hydrolysate. Cheng et al. (2014) also reported a Y_Et/TotS_ of 0.42 g_Et_/g_Glu_ from non-detoxified CC hydrolysate with *C. tropicalis* W103 as a fermenting microbe. *C. magnoliae* TISTR 5664 in this study could significantly degrade inhibitors formed during the pretreatment step. Evidently, this strain indicated the similar tolerant ability as *C. tropicalis* W103 which could totally degrade HMF and furfural after 60 h while acetic acid was consumed by 89.4% after xylitol production. This resulted in a relatively lower Y_Xy/Xyl_ of 0.32 g_Xy_/g_Xyl_ using non-detoxified CC hydrolysate (Cheng et al., 2014). Seemingly, the detoxification step was, thus, unnecessary for cost-saving purposes, as demonstrated by the current study and Cheng et al. (2014).

The report published by Nunta et al. (2023) indicated that *C. tropicalis* was the statistically significant highest (*p* ≤ 0.05) ethanol producer with a lower Y_Et/TotS_ of 0.38 g_Et_/g_TotS_ (15.3 g/L). In fact, *C. tropicalis* and *C. magnoliae* could produce the highest xylitol and ethanol concentrations as evident from previous studies by our group while the ability to produce xylitol was lacking in *S. cerevisiae* (Cunha et al., 2019). To improve the xylitol and ethanol yields, the corresponding production processes were carried out under microaerobic and partially anaerobic conditions, respectively, using *C. magnoliae*. The [PAC] in the overall phases were achieved at a similar level between the mixture of *C. magnoliae* whole cells derived from xylitol and ethanol production steps and *C. tropicalis* whole cells from the ethanol production step. In term of PAC activity, *C. magnoliae* whole cells obtained from the xylitol production step had the twice induced PDC activity level than those derived from the ethanol production step.

Even though a two-phase emulsion system could improve PAC production due to its compatibility with the hydrophobic structure of organic phase (Sandford et al., 2005), the associated cost per unit of PAC production could be higher if the produced PAC was not sufficiently high enough to offset the cost of employed organic phase. This was in agreement with the work of Kumar et al. (2023) where the total production cost between a two-phase emulsion system with a volume ratio of 1:1 (vegetable oil:1 M Pi buffer) was increased by 135% in comparison with a single-phase emulsion system (USD 1.93/kg PAC compared with USD 0.82/kg PAC). This was nearly equivalent to 146% increase to the PAC being formed which might not be worthwhile to the investment cost. In fact, the total cost of two-phase emulsion system with vegetable oil and 1 M Pi buffer in this study was much lower (USD 0.42/kg PAC) when the optimal volume ratio of 0.43:1 was employed (Gunawan et al., 2008). The cost effectiveness of this system was significantly pronounced (*p* ≤ 0.05) when compared to the work of Kumar et al. (2023) with a cost mitigation of 78.4% and 49.1% for similar two-phase emulsion and single-phase emulsion systems. The potential of the multi-pass recycling system of vegetable oil as predicted by Kumar et al. (2023) was quite attractive to further the lowering in production cost while facilitating PAC accumulation. The expected cost reduction in this system could be up to 30% in the third-pass biotransformation with relatively higher [PAC].

## 5 Conclusion

The optimal conditions of pretreatment strategy utilizing diluted [sulfuric acid] in boiling water and the subsequent enzymatic hydrolysis step of SCB, RS, and CC were elucidated and could be adopted as the conditions of choice for the industrial-scale production. The relatively high-valued chemicals such as xylitol, ethanol, and PAC could be produced to assure an overall economically competitive process with the wild-type *C. magnoliae* TISTR 5664. The ability of this yeast to degrade a statistically significant amount of HMF, acetic acid, and furfural during xylitol production from CC xylose-rich hydrolysate was noted in our study without the necessity of adding the detoxification step. In future study, the production of xylitol and ethanol with the implementation of cell recycling will be investigated in both CC xylose-rich and CC glucose-rich hydrolysates. The multi-pass recycling procedure of organic phase in the two-phase emulsion biotransformation system for PAC biotransformation will also be evaluated.

## Figures and Tables

**Table:** 

**[ ]**	Concentration
**μ**_**max**_	Maximum specific growth rate (h^−1^)
**aq**	Aqueous
**Bz**	Benzaldehyde
**CBU**	Cellobiase activity unit
**CC**	Corn cob
**CCD**	Central composite design
**Et**	Ethanol
**FPU**	Filter paper unit
**Glu**	Glucose
**H**_**2**_ **SO**_**4**_	Sulfuric acid
**LSR**	Liquid-to-solid ratio
**MOPS**	3-(N-Morpholino)propanesulfonic acid
**MV**	Mean value
**org**	Organic
**PAC**	Phenylacetylcarbinol
**PDC**	Pyruvate decarboxylase
**Pi**	Phosphate
**Pyr**	Sodium pyruvate
**Q**_**Et,max**_	Volumetric productivity of ethanol per liter per h (g_Et_/L/h)
**Q**_**Xy,max**_	Volumetric productivity of xylitol per liter per h (g_Xy_/L/h)
**Q**_**PAC**_	Volumetric productivity of PAC per liter per h (mM/h)
**q**_**Et,max**_	Maximum specific ethanol production rate (g_Et_/g_X_/h)
**q**_**TotS,max**_	Maximum specific total sugars consumption rate (g_TotS_/g_X_/h)
**q**_**Xy,max**_	Maximum specific xylitol production rate (g_Xy_/g_X_/h)
**RS**	Rice straw
**RSM**	Response surface methodology
**SCB**	Sugarcane bagasse
**SE**	Standard errors
**SE**_**max**_	The highest SE among the two adjacent MVs of subsequent time courses
**S**_**p,PAC**_	_Specific PAC production_ (mg/U_ICA_)
**TISTR**	Thailand Institute of Scientific and Technological Research
**TotS**	Total sugars
**U**_**ICA**_	Initial carboligase activity unit
**X**	Dried biomass
**Xy**	Xylitol
**Xyl**	Xylose
**Y**_**Et/TotS**_	Yield of ethanol produced over total sugars consumed (g_Et_/g_TotS_)
**Y**_**X/TotS**_	Yield of dried biomass produced over total sugars consumed (g_X_/g_TotS_)
**Y**_**Xy/Xyl**_	Yield of xylitol produced over xylose consumed (g_Xy_/g_Xyl_)
**Y**_**PAC/Bz**_	Yield of PAC produced over Bz consumed (mol_PAC_/mol_Bz_)
**Y**_**PAC/Pyr**_	Yield of PAC produced over Pyr consumed (mol_PAC/_mol_Pyr_)
**YMX**	Yeast-malt medium supplemented with 5 g/L xylose

## Data Availability

The raw data supporting the conclusion of this article will be made available by the authors, without undue reservation.
