# Supplementary material for: Pretreatment and enzymatic hydrolysis optimization of lignocellulosic biomass for ethanol, xylitol, and phenylacetylcarbinol co-production using Candida magnoliae
Source: Front Bioeng Biotechnol. 2024 Jan 18;11:1332185. doi: 10.3389/fbioe.2023.1332185 (PMC10830760; doi:10.3389/fbioe.2023.1332185)
Supplement: Supplementary file 1 [file Table1.DOCX]

Supplementary Material

# Supplementary Tables

**Supplementary Table S1.** Experimental variables with corresponding ranges and levels of diluted [sulfuric acid] pretreatment optimization based on the CCD.

| Variables | Symbol code | Range and levels | | |
| --- | --- | --- | --- | --- |
|  |  | -1 | 0 | +1 |
| [Sulfuric acid] (% w/v) | *X*_1_ | 0.50 | 2.75 | 5.00 |
| Reaction time (min) | *X*_2_ | 30 | 135 | 240 |

**Supplementary Table S2.** Experimental variables with corresponding ranges and levels of enzyme hydrolysis optimization based on the CCD.

| Variables | Symbol code | Ranges and levels | | |
| --- | --- | --- | --- | --- |
|  |  | -1 | 0 | +1 |
| [Pretreated solid] (% w/v) | *X*_a_ | 5 | 12.5 | 20 |
| Hydrolysis time (h) | *X*_b_ | 48 | 144 | 240 |

**Supplementary Table S3.** ANOVA of the proposed models for cellulose and lignin content of SCB in diluted [sulfuric acid] pretreatment optimization.

| **Source** | **SS** | **DF** | **MS** | ***F*-value** | ***p*-value** | **Significant** |
| --- | --- | --- | --- | --- | --- | --- |
| **Cellulose content (*Y*_1,SCB_) (quadratic model)** | | | | | | |
| Model | 102.95 | 5 | 20.59 | 26.46 | 0.0002 | **significant** |
| *X*_1_ | 72.29 | 1 | 72.29 | 92.91 | < 0.0001 |  |
| *X*_2_ | 22.60 | 1 | 22.60 | 29.05 | 0.0010 |  |
| *X*_1_^2^ | 7.92 | 1 | 7.92 | 10.18 | 0.0153 |  |
| *X*_2_^2^ | 0.64 | 1 | 0.64 | 0.83 | 0.3930 |  |
| *X*_1_*X*_2_ | 0.048 | 1 | 0.048 | 0.061 | 0.8116 |  |
| Residual | 5.45 | 7 | 0.78 |  |  |  |
| Lack of Fit | 3.21 | 3 | 1.07 | 1.92 | 0.2679 | **not significant** |
| Pure Error | 2.23 | 4 | 0.56 |  |  |  |
| Cor Total | 108.40 | 12 |  |  |  |  |
| **Lignin content (*Y*_2,SCB_) (quadratic model)** | | | | | | |
| Model | 22.06 | 5 | 4.41 | 22.44 | 0.0004 | **significant** |
| *X*_1_ | 13.72 | 1 | 13.72 | 69.79 | < 0.0001 |  |
| *X*_2_ | 6.81 | 1 | 6.81 | 34.66 | 0.0006 |  |
| *X*_1_^2^ | 0.57 | 1 | 0.57 | 2.90 | 0.1324 |  |
| *X*_2_^2^ | 0.38 | 1 | 0.38 | 1.92 | 0.2083 |  |
| *X*_1_*X*_2_ | 3.621E-003 | 1 | 3.621E-003 | 0.018 | 0.8959 |  |
| Residual | 1.38 | 7 | 0.20 |  |  |  |
| Lack of Fit | 1.03 | 3 | 0.34 | 4.01 | 0.1065 | **not significant** |
| Pure Error | 0.34 | 4 | 0.086 |  |  |  |
| Cor Total | 23.44 | 12 |  |  |  |  |

SS = sum of squared, DF = degree of freedom, MS = mean square

**Supplementary Table S4.** Analysis of variance of the proposed models for cellulose and lignin content of RS in diluted [sulfuric acid] pretreatment optimization.

| **Source** | **SS** | **DF** | **MS** | ***F*-value** | ***p*-value** | **Significant** |
| --- | --- | --- | --- | --- | --- | --- |
| **Cellulose content (*Y*_1,RS_) (quadratic model)** | | | | | | |
| Model | 422.80 | 5 | 84.56 | 55.78 | < 0.0001 | **significant** |
| *X*_1_ | 295.47 | 1 | 295.47 | 194.91 | < 0.0001 |  |
| *X*_2_ | 30.99 | 1 | 30.99 | 20.44 | 0.0027 |  |
| *X*_1_^2^ | 49.28 | 1 | 49.28 | 32.51 | 0.0007 |  |
| *X*_2_^2^ | 13.39 | 1 | 13.39 | 8.83 | 0.0208 |  |
| *X*_1_*X*_2_ | 0.14 | 1 | 0.14 | 0.092 | 0.7707 |  |
| Residual | 10.61 | 7 | 1.52 |  |  |  |
| Lack of Fit | 7.97 | 3 | 2.66 | 4.01 | 0.1063 | **not significant** |
| Pure Error | 2.65 | 4 | 0.66 |  |  |  |
| Cor Total | 433.41 | 12 |  |  |  |  |
| **Lignin content (*Y*_2,RS_) (quadratic model)** | | | | | | |
| Model | 30.68 | 5 | 6.14 | 49.61 | < 0.0001 | **significant** |
| *X*_1_ | 19.43 | 1 | 19.43 | 157.07 | < 0.0001 |  |
| *X*_2_ | 6.96 | 1 | 6.96 | 56.28 | 0.0001 |  |
| *X*_1_^2^ | 1.99 | 1 | 1.99 | 16.08 | 0.0051 |  |
| *X*_2_^2^ | 0.21 | 1 | 0.21 | 1.71 | 0.2320 |  |
| *X*_1_*X*_2_ | 1.14 | 1 | 1.14 | 9.20 | 0.0190 |  |
| Residual | 0.87 | 7 | 0.12 |  |  |  |
| Lack of Fit | 0.66 | 3 | 0.22 | 4.24 | 0.0982 | **not significant** |
| Pure Error | 0.21 | 4 | 0.052 |  |  |  |
| Cor Total | 31.55 | 12 |  |  |  |  |

SS = sum of squared, DF = degree of freedom, MS = mean square

**Supplementary Table S5.** Analysis of variance of the proposed models for cellulose and lignin content of CC in diluted [sulfuric acid] pretreatment optimization.

| **Source** | **SS** | **DF** | **MS** | ***F*-value** | ***p*-value** | **Significant** |
| --- | --- | --- | --- | --- | --- | --- |
| **Cellulose content (*Y*_1,CC_) (quadratic model)** | | | | | | |
| Model | 714.12 | 5 | 142.82 | 143.63 | < 0.0001 | **significant** |
| *X*_1_ | 384.27 | 1 | 384.27 | 386.45 | < 0.0001 |  |
| *X*_2_ | 163.62 | 1 | 163.62 | 164.55 | < 0.0001 |  |
| *X*_1_^2^ | 102.54 | 1 | 102.54 | 103.12 | < 0.0001 |  |
| *X*_2_^2^ | 11.90 | 1 | 11.90 | 11.96 | 0.0106 |  |
| *X*_1_*X*_2_ | 1.25 | 1 | 1.25 | 1.25 | 0.2999 |  |
| Residual | 6.96 | 7 | 0.99 |  |  |  |
| Lack of Fit | 5.43 | 3 | 1.81 | 4.73 | 0.0838 | **not significant** |
| Pure Error | 1.53 | 4 | 0.38 |  |  |  |
| Cor Total | 721.08 | 12 |  |  |  |  |
| **Lignin content (*Y*_2,CC_) (quadratic model)** | | | | | | |
| Model | 30.68 | 5 | 6.14 | 49.61 | < 0.0001 | **significant** |
| *X*_1_ | 19.43 | 1 | 19.43 | 157.07 | < 0.0001 |  |
| *X*_2_ | 6.96 | 1 | 6.96 | 56.28 | 0.0001 |  |
| *X*_1_^2^ | 1.99 | 1 | 1.99 | 16.08 | 0.0051 |  |
| *X*_2_^2^ | 0.21 | 1 | 0.21 | 1.71 | 0.2320 |  |
| *X*_1_*X*_2_ | 1.14 | 1 | 1.14 | 9.20 | 0.0190 |  |
| Residual | 0.87 | 7 | 0.12 |  |  |  |
| Lack of Fit | 0.66 | 3 | 0.22 | 4.24 | 0.0982 | **not significant** |
| Pure Error | 0.21 | 4 | 0.052 |  |  |  |
| Cor Total | 31.55 | 12 |  |  |  |  |

SS = sum of squared, DF = degree of freedom, MS = mean square

**Supplementary Table S6.** Analysis of variance of the proposed models for glucose yield of SCB in enzymatic hydrolysis optimization.

| **Source** | **SS** | **DF** | **MS** | ***F*-value** | ***p*-value** | **Significant** |
| --- | --- | --- | --- | --- | --- | --- |
| **Glucose yield (*Y*_a,SCB_) (quadratic model)** | | | | | | |
| Model | 246.11 | 5 | 49.22 | 69.00 | < 0.0001 | **significant** |
| *X*_a_ | 179.93 | 1 | 179.93 | 252.22 | < 0.0001 |  |
| *X*_b_ | 23.01 | 1 | 23.01 | 32.25 | 0.0008 |  |
| *X*_a_^2^ | 22.94 | 1 | 22.94 | 32.15 | 0.0008 |  |
| *X*_b_^2^ | 0.78 | 1 | 0.78 | 1.10 | 0.3293 |  |
| *X*_a_*X*_b_ | 11.65 | 1 | 11.65 | 16.33 | 0.0049 |  |
| Residual | 4.99 | 7 | 0.71 |  |  |  |
| Lack of Fit | 4.05 | 3 | 1.35 | 5.76 | 0.0619 | **not significant** |
| Pure Error | 0.94 | 4 | 0.23 |  |  |  |
| Cor Total | 251.10 | 12 |  |  |  |  |

SS = sum of squared, DF = degree of freedom, MS = mean square

**Supplementary Table S7.** Analysis of variance of the proposed models for glucose yield of RS in enzymatic hydrolysis optimization.

| **Source** | **SS** | **DF** | **MS** | ***F*-value** | ***p*-value** | **Significant** |
| --- | --- | --- | --- | --- | --- | --- |
| **Glucose yield (*Y*_a,RS_) (quadratic model)** | | | | | | |
| Model | 470.46 | 5 | 94.09 | 35.18 | < 0.0001 | **significant** |
| *X*_a_ | 450.58 | 1 | 450.58 | 168.49 | < 0.0001 |  |
| *X*_b_ | 0.44 | 1 | 0.44 | 0.16 | 0.6977 |  |
| *X*_a_^2^ | 3.24 | 1 | 3.24 | 1.21 | 0.3074 |  |
| *X*_b_^2^ | 9.21 | 1 | 9.21 | 3.45 | 0.1058 |  |
| *X*_a_*X*_b_ | 3.396E-003 | 1 | 3.396E-003 | 1.270E-003 | 0.9726 |  |
| Residual | 18.72 | 7 | 2.67 |  |  |  |
| Lack of Fit | 14.46 | 3 | 4.82 | 4.53 | 0.0893 | **not significant** |
| Pure Error | 4.26 | 4 | 1.06 |  |  |  |
| Cor Total | 489.18 | 12 |  |  |  |  |

SS = sum of squared, DF = degree of freedom, MS = mean square

**Supplementary Table S8.** Analysis of variance of the proposed models for glucose yield of CC in enzymatic hydrolysis optimization.

| **Source** | **SS** | **DF** | **MS** | ***F*-value** | ***p*-value** | **Significant** |
| --- | --- | --- | --- | --- | --- | --- |
| **Glucose yield (*Y*_a,CC_) (quadratic model)** | | | | | | |
| Model | 1130.01 | 5 | 226.00 | 65.26 | < 0.0001 | **significant** |
| *X*_a_ | 954.06 | 1 | 954.06 | 275.50 | < 0.0001 |  |
| *X*_b_ | 3.74 | 1 | 3.74 | 1.08 | 0.3331 |  |
| *X*_a_^2^ | 79.18 | 1 | 79.18 | 22.87 | 0.0020 |  |
| *X*_b_^2^ | 2.19 | 1 | 2.19 | 0.63 | 0.4529 |  |
| *X*_a_*X*_b_ | 65.29 | 1 | 65.29 | 18.85 | 0.0034 |  |
| Residual | 24.24 | 7 | 3.46 |  |  |  |
| Lack of Fit | 17.48 | 3 | 5.83 | 3.45 | 0.1314 | **not significant** |
| Pure Error | 6.76 | 4 | 1.69 |  |  |  |
| Cor Total | 1154.25 | 12 |  |  |  |  |

SS = sum of squared, DF = degree of freedom, MS = mean square
